# National Medical Convention—For Revising the Pharmacopœia of the United States

**Published:** 1850-06

**Authors:** 


*National Medical Convention—For revising the Pharmacopœia of the United States.*

The fourth decennial convention for revising the Pharmacopœia of the United Satates met at Washington on Monday, the 6th instant. The following delegates were present in the convention :

From Rhode Island Medical Society, Dr. Joseph Mauran.

From Geneva Medical College, Dr. James Bryan.

From the College of Pharmacy of the City of New York, Messrs. John Milhau and George D. Coggeshall.

From the Medical Society of New Jersey, Drs. Lewis Condict and Wm. A. Newell.

From the College of Physicians of Philadelphia, Drs. Joseph Carson, Henry Bond, and Francis West.

From the University of Pennsylvania, Dr. George B. Wood and James B. Rogers.

From the Jefferson Medical College of Philadelphia, Dr. Franklin Bache.

From the Medical Faculty of the Peennsylvania College, Dr. H. S. Patterson.

From the Medico-Chirurgical College of Philadelphia, Dr. Clinton G. Stees.

From the Philadelphia College of Pharmacy, Messrs. D. B. Smith, Charles Ellis, and Wm. Proctor, Jr.

From the Medical Society of Delaware, Drs. Isaac Jump and J. W. Thomson.

From the Medical and Chirurgical Faculty of Maryland, Drs. David Stewart and Joshua I. Cohen.

From the Medical Society of the District of Columbia, Drs. J. C. Hall and Harvey Lindsly.

From the National Medical College of the District of Columbia, Drs. Joshua Riley, Thomas Miller, and Edward Foreman.

From the Medical Department of the National Institute, D. C. Drs. Jas. Wynne and S. D. Gale.

From the Georgetown Medical College, Dr. F. Howard.

And from the Rush Medical College, Illinois, Dr. G. N. Fitch.

The credentials of delegates from the New Hampshire Medical Institution, the University of Buffalo, the Medical Department of Hampden Sidney College, the Medical Society of South Carolina, the Medical College of Ohio, the Cincinnati College of Pharmacy, the Missouri Medical Society, and the Medical Faculty of the University of Iowa, were presented by the Vice President of the Convention of 1840 ; but these delegates did not make their appearance during the session of the convention.

A temporary organization was effected by calling Dr. Lewis Condict, President of the convention of 1840, to the chair, and appointing Dr. Harvey Liudsly, Secretary. A committee of five was then appointed, consisting of Dr. Bache, Dr. Mauran, Dr. Thomson, Dr. Miller, and Mr. Coggshall, to nominate permanent officers of the convention, with instructions to name two Vice Presidents, instead of one, as had beeen the custom on former occasions. This committee retired, and, after a short consultation, reported the names of the following delegates, viz :

For President. Dr. George B. Wood, of Pennsylvania.

For Vice Presidents, Dr. Joseph Mauran, of Rhode Island, and Dr. D. Y. Simons, South Carolina.

For Secretary, Dr. Harvey Lindsly, of the District of Columbia ; and for Assistant Secretary, Dr. Edward Foreman, of the same place.

The nominations were confirmed by the convention, and the President took the chair.

On motion, it was

*Resolved*, That the Surgeon-General of the Army, the Chief of the Naval Bureau of medicine and Surgery, and such of the members of the two

Houses of Congress as might be medical graduates, should be invited to take seats in the convention, and participate in its proceedings.

In conformity with the directions of the preceding convention, the committee of revision and publication, appointed by that body, presented a report of their proceedings, which was accepted.

The delegates of the several medical bodies represented in the convention were then called on for contributions towards the revision of the Pharmacopœia ; when reports were handed in from the delegate of the Rhode Island Medical Society, from the College of Pharmacy of the city of New York, from the College of Physicians of Philadelphia, from the Philadelphia College of Pharmacy, and from the medical and Chirurgical Faculty of Maryland. These were referred to a committee, consisting of Dr. Bond, Dr. Mauran, Dr. Cohen, Dr. Miller, and Mr. Milhau, with directions to report a plan for the revision and publication of the Pharmacopœia ; after which the convention adjourned to the following day.

At the next morning, on Tnesday morning, a committee was appointed to examine the accounts and vouchers presented by the committee of revision and publication of the preceding convention, and reported that they had found them correct.

Dr. Bond, from the committee to which had been referred the reports from various medical bodies represented in the convention, reported the following resolution:

1. That a committee of revision and publication, consisting of nine members, be appointed, to which shall be referred all communications offered to the convention in relation to the revision of the Pharmacopœia, and that three of this committee shall form a quorum.

2. That the committee shall meet in the city of Philadelphia, and be convened as soon as practicable by the chairman.

3. That said committee shall be authorized to publish the work of revision, and to take all other measures which may be necessary to carry out the views and intentions of the convention.

4. That the commitiee shall have power to fill its own vacancies.

5 That, after the completion of its labors, the committee shall submit a report of its proceedings to the Secretary of this convention, to be held before the next convention.

The resolutions were adopted, and the following delegates appointed on on the committee, viz: Dr. Franklin Bache, Dr. Joseph Carson, and Wm. Proctor, Jr. of Philadelphia; Dr. Joseph Mauran, of Providence Rhode Island; Mr. John Milhau, of the city of New York; Dr J. W. Thomson, of Wilmington, Delaware; Dr. David Stewart, of Baltimore; Dr. Joshua Riley, of the District of Columbia; and Dr. G. N. Fitch, of Logansport, Indiana.

It was resolved that the President of the convention be added to the above committee, and serve as its chairman.

In reference to the manner of calling and the mode of constituting the next decennial convention, to meet in the year 1860, it was

*Resolved,* That the regulations in reference to the present convention, adopted by that of the year 1840, and published in the last edition of Pharmacopœia, should be adopted, with the necessory modifications in relation to the dates; the day of meeting being changed from the first Monday to the first Wednesday in May

A letter was read inviting the members of the convention to a dinner, to be given at the National Hotel, by the medical gentleman of Washington. The invitation was accepted, and the thanks of the convention voted to the gentlemen referred to for their hospitality.

The thanks of the convention were also unanimously voted to Dr. Lewis Condict, President of the last convention, for valuable services; and to the Board of Aldermen, of the city of Washington, for their courtesy in offering their hall for the sittings of this convention.

The convention then adjourned.

After the adjournment, Dr. Wm. B. Chapman, one of the delegates from Cincinnati College of Pharmacy, arriving in Washington, stated to the Secretary his concurrence in the proceedings of the convention.

HARVEY LINDSLY, M. D.
*Secretary of the Convention.*

Washington, may 9th 1850

